# Skimmed Milk an Important Adjunct in the Treatment of Catarrh of the Bladder and Cystitis

**Published:** 1877-11

**Authors:** 


Skimmed Milk an Important Adjunct in the Treatment of Catarrh of the Bladder and Cystitis.—Gonnorrhœa is the legitimate parent of catarrh of the bladder, as well as the cause of cystitis. Though the terms are not synonymous, yet they are identical, being indicative of different stages of the same disease, inflammation of the bladder. I have had many cases of catarrh, consequent upon protracted gonnorrhœal attacks, causing stricture, and thereby inducing catarrh. The symptoms of either catarrh of the bladder or cystitis are more violent and distressing than an organic stricture, and equally as painful as an urinary calculus. The strangury is intense, and as long as the ureters convey urine into the bladder, so long does the scalding pain continue. Micturition affords only temporary relief, and really intensifies the desire to urinate, for if a large amount of urine could be retained in the bladder, the ammoniacal decomposition would virtually dilute the influx, and the bladder thus acquire a tolerance of the contained urine, mingled with the mucoid secretion. The metallic sound can only decide satisfactorily as to the presence or not of urinary calculus, because the chain of subjective symptoms is so simple in cystitis, stricture and stone, that to be perfectly safe in your diagnosis the sound had better be used to differentiate. Stone, however, is more to be apprehended in limestone countries than in the southern portion of the United States.

In my practice, excluding the presence of an urinary calculus as a primitive source of catarrh or cystitis, I must accredit to gonorrhœa the causation of catarrh, cystitis and stricture, primarily in some cases and secondarily in others. I have had patients consult me for stricture when the largest bougie could be passed; yet upon a close catechism and examination of the urine, I have found them suffering with the most excruciating agony from catarrh of the bladder and not from stricture of the urethra. In fact, they had been treated for stricture up to the date of application to me for relief. I have thrown aside instruments and put them upon skimmed milk, with the following prescription:

| | | |
|---|---|---|
| ℞.—Quiniæ hypophosphitis, | . . . | ʒ ss |
| Ferri pyrophosphatis, | . . . . . | ʒ ss |
| Pulv. ergotini (or Bonjean's), | . . | gr. xv |
| Ext. nucis vomicæ, | . . . . . | gr. vii |

M. ft. pil. No. xv. Sig. one to be taken every four hours.

The above, in addition to skimmed milk, has invariably been successful in a comparatively brief period of time. Skimmed milk has been used frequently enough to decide its peculiar specific virtues, independently of other internal treatment, which treatment of course is clearly indicated. In these cases I have abandoned the vaunted astringent injections into the bladder, for the more pleasant and readily administered medicine skimmed milk, and can cheerfully confirm all that has been written of its influence in albuminuria as applicable to cases of catarrh of the bladder and cystitis, the latter when not of too long standing.—*American Practitioner.*

